# Global translational landscape of the *Candida albicans* morphological transition

**DOI:** 10.1093/g3journal/jkaa043

**Published:** 2020-12-24

**Authors:** Vasanthakrishna Mundodi, Saket Choudhary, Andrew D Smith, David Kadosh

**Affiliations:** 1 Department of Microbiology, Immunology and Molecular Genetics, University of Texas Health Science Center at San Antonio, San Antonio, TX 78229, USA; 2 Department of Computational Biology and Bioinformatics, University of Southern California, Los Angeles, CA 90089, USA

**Keywords:** *Candida albicans*, morphology, ribosome profiling, translational regulation, fungal pathogenesis

## Abstract

*Candida albicans*, a major human fungal pathogen associated with high mortality and/or morbidity rates in a wide variety of immunocompromised individuals, undergoes a reversible morphological transition from yeast to filamentous cells that is required for virulence. While previous studies have identified and characterized global transcriptional mechanisms important for driving this transition, as well as other virulence properties, in *C. albicans* and other pathogens, considerably little is known about the role of genome-wide translational mechanisms. Using ribosome profiling, we report the first global translational profile associated with *C. albicans* morphogenesis. Strikingly, many genes involved in pathogenesis, filamentation, and the response to stress show reduced translational efficiency (TE). Several of these genes are known to be strongly induced at the transcriptional level, suggesting that a translational fine-tuning mechanism is in place. We also identify potential upstream open reading frames (uORFs), associated with genes involved in pathogenesis, and novel ORFs, several of which show altered TE during filamentation. Using a novel bioinformatics method for global analysis of ribosome pausing that will be applicable to a wide variety of genetic systems, we demonstrate an enrichment of ribosome pausing sites in *C. albicans* genes associated with protein synthesis and cell wall functions. Altogether, our results suggest that the *C. albicans* morphological transition, and most likely additional virulence processes in fungal pathogens, is associated with widespread global alterations in TE that do not simply reflect changes in transcript levels. These alterations affect the expression of many genes associated with processes essential for virulence and pathogenesis.

## Teaser

*Candida albicans* undergoes a reversible transition from single budding yeast to filamentous cells that is required for virulence. Here, Mundodi *et al* use ribosome profiling to determine the first global translational profile associated with the *C. albicans* morphological transition. They find that many important virulence factors strongly induced at the transcriptional level show significantly reduced translational efficiency. Overall, their findings suggest that key virulence processes in *C. albicans*, and most likely other fungal pathogens, are under widespread translational control.

## Introduction

*Candida albicans* is normally found as a commensal in most healthy individuals. However, in immunocompromised individuals such as AIDS patients, organ transplant recipients, and cancer patients. *Candida albicans* is a major human fungal pathogen responsible for a wide variety of both systemic and mucosal infections (Shepherd *et al.* 1985; Odds 1988, 1994; Dupont 1995; Weig *et al.* 1998). Approximately 70% of all women will experience at least one episode of vaginal candidiasis in their lifetime and *Candida* species represent the fourth-leading cause of hospital-acquired bloodstream infections in the U.S. with a mortality rate between 35% and 60% (Edmond *et al.* 1999; Wisplinghoff *et al.* 2004; Sobel 2007). Despite the high morbidity and mortality rates, there are only three major classes of antifungals available for the treatment of *Candida* infections and about $1 billion per year is spent on these treatments (Miller *et al.* 2001; Revie *et al.* 2018).

In order to design more effective antifungal strategies, it is important to obtain a comprehensive understanding of *C. albicans* virulence properties as well as genome-wide gene expression patterns that are involved in controlling these properties. The ability to undergo a morphological transition from single budding yeast cells to pseudohyphal and hyphal filaments (elongated cells attached end-to-end) represents one of the most important *C. albicans* virulence traits (Mitchell 1998; Calderone and Fonzi 2001; Brown 2002; Saville *et al.* 2003; Kumamoto and Vinces 2005). This reversible transition is induced in response to a wide variety of environmental conditions in the host environment, including serum, body temperature (37°C) and neutral/alkaline pH (Brown 2002; Biswas *et al.* 2007). *C. albicans* filaments contribute to virulence by promoting the invasion of a variety of host tissues, including both epithelial and endothelial cell layers as well as lysis of macrophages (Kumamoto and Vinces 2005). Filamentous growth is also important for thigmotropism (contact sensing), which is thought to promote *C. albicans* invasion of points of weakened tissue integrity (Sherwood *et al.* 1992).

Previous studies have identified a variety of signal transduction pathways important for driving the *C. albicans* yeast-filament transition in response to host environmental cues, including MAP kinase, Ras-cAMP-PKA, and pH-mediated pathways (Ernst 2000; Biswas *et al.* 2007; Kadosh 2017). These signaling pathways, in turn, promote activation of a diverse array of transcriptional regulators which drive the expression of genes involved in filamentation; many of these regulators are known to control one another through complex interaction networks (Ernst 2000; Biswas *et al.* 2007; Kadosh 2017). Global transcriptional profiling studies of the *C. albicans* morphological transition in response to the growth in serum at 37°C (one of the strongest filament-inducing conditions) have revealed that in addition to genes involved in the physical process of filamentation, genes associated with a variety of other virulence properties, including adhesion to host cells, degradation of host cell membrane proteins, and the response to environmental stresses, are strongly induced (Nantel *et al.* 2002; Kadosh and Johnson 2005; Bruno *et al.* 2010). Genes involved in bipolar budding, cell division, ER-Golgi transport, and secretion are also induced during *C. albicans* morphogenesis.

While both transcriptional and post-translational mechanisms that regulate the *C. albicans* morphological transition have been well-studied, considerably less is known about the role of translational mechanisms in controlling this transition, as well as virulence properties and other biological processes in *C. albicans* and other pathogenic fungi. However, a few recent studies have begun to shed light in this area. More specifically, *UME6*, which encodes a key transcriptional regulator of *C. albicans* filamentous growth and pathogenesis, was shown to be controlled by a 5ʹ UTR-mediated translational efficiency (TE) mechanism (Childers *et al.* 2014); importantly, the level of *UME6* translational inhibition is modulated by a variety of filament-inducing conditions similar to those encountered in the host. Subsequent studies also demonstrated a role for 5ʹ UTR-mediated TE mechanisms in controlling *WOR1*, which encodes a key regulator of *C. albicans* white-opaque switching and mating, as well as *EFG1*, encoding another filamentous growth transcriptional regulator (Guan and Liu 2015; Desai *et al.* 2018). These 5ʹ UTRs could function to regulate translation of their target genes through a variety of mechanisms, including the formation of strong secondary structures and association with RNA-binding proteins to inhibit ribosome accessibility, upstream open reading frames (uORFs) as well as alternative transcript localization via zip code sequences (Jansen 2001; Mignone *et al.* 2002; Pickering and Willis 2005; Adeli 2011; Araujo *et al.* 2012). Indeed, a well-characterized uORF-mediated mechanism has been shown to control translation of *GCN4*, an important regulator of the amino acid starvation response, in both *S. cerevisiae* and *C. albicans* (Hinnebusch and Natarajan 2002; Sundaram and Grant 2014). In addition, the Dom34 RNA-binding protein has been shown to promote the translation of genes important for *C. albicans* protein *O*-mannosylation via the 5ʹ UTR (van Wijlick *et al.* 2016). In the human fungal pathogen, *Cryptococcus neoformans*, a mechanism involving phosphorylation of the translation factor eIF2α was found to be important for inhibiting protein synthesis in response to oxidative stress (Leipheimer *et al.* 2019). Finally, treatment of the emerging fungal pathogen *Candida auris* with rocaglate drugs was demonstrated to inhibit translation by targeting the eIF4A translation initiation factor and promoting the activation of a cell death program (Iyer *et al.* 2020).

While the studies described above highlight the importance of individual translational mechanisms in controlling virulence properties of human fungal pathogens, considerably little is known about global translational changes in gene expression that are associated with these properties. Here, we optimize ribosome profiling (Ingolia *et al.* 2009) for *C. albicans* and use this powerful approach to determine the genome-wide global translational profile during the *C. albicans* morphological transition. Interestingly, we find that many genes known to be strongly induced at the transcript level during this transition, which is associated with filamentation, pathogenesis, and the response to host environmental stresses, show significantly reduced TE. Using recently developed bioinformatics methods for the analysis of ribosome profiling data, we also identify, on a global scale, ribosome pausing sites as well as potential uORFs and novel ORFs, several of which appear to be differentially translated during the *C. albicans* yeast-filament transition. These findings provide new insight into the global translational landscape of a major human fungal pathogen and suggest that in addition to changes in transcript levels, the *C. albicans* morphological transition is also associated with widespread alterations in TE.

## Materials and methods

### Cell growth and extract preparation

Serum inductions were carried out in yeast extract peptone dextrose (YEPD) medium as described previously using *C. albicans* wild-type strain DK318 (Banerjee *et al.* 2008) with the following exceptions: (1) the initial starting overnight culture volume was 150 mL and overnight cultures were diluted at OD_600_ ∼4.0, (2) cells were diluted into a final culture volume of 450 mL of YEPD at 30°C or YEPD + 10% serum at 37°C. Cells were harvested at the 1-h post-induction time point and recovered by rapid filtration without pretreatment using a protein synthesis inhibitor. Next, cells were scraped off the filter paper with a cell scraper, placed in ice-cold lysis buffer (1× yeast polysome buffer (Illumina), 1% Triton X-100 (Illumina), 50 µg/mL GMPPNP (Sigma), 10 µg/mL Blasticidin S (InvivoGen)) and homogeneously mixed prior to snap freezing in liquid nitrogen; please note that the protein synthesis inhibitor, Blasticidin S, was added post-cell harvest as described previously (Muzzey *et al.* 2014). Lysates were prepared as described previously (Spealman *et al.* 2016) with a few modifications. Flash frozen cells were thawed in an ice water slurry and cell suspensions were transferred into tubes with 0.5 mm diameter acid-washed glass beads and placed on ice for 5 min. The samples were lysed by vortexing eight times for 30 s each with a 30-s rest on ice between each vortex. Samples were further processed according to instructions for the Illumina TrueSeq Ribo Profile (Yeast) Kit. Briefly, lysates were precleared by centrifugation at 3000 g for 5 min at 4°C to remove cell debris and further clarified by centrifugation at 20,000 g for 10 min at 4°C. The lysates were treated for 10 min on ice with 10 U/mL DNase I (Illumina) and quantified by measuring absorbance at 260 nm (A260). Finally, 100 µL aliquots were frozen in liquid nitrogen and stored at −80°C until further use.

### Ribosome profiling

Ribosome profiling and library preparation were carried out according to the protocol described for the TrueSeq Ribo Profile (Yeast) Kit (Illumina) with several modifications. Lysates were digested with 15 U RNase I (Ambion) per A260 unit and incubated at room temperature for 45 min with gentle rotation. The reactions were stopped by the addition of 10 µL (10 U/µL) SUPERase-IN (Ambion). 80S monosome fractions were purified from the cell lysate using MicroSpin S-400 HR columns (GE Healthcare). Ribosome protected fragments (RPFs) were further purified using the RNA Clean & Concentrator-25 Kit (Zymo Research) method. This kit was also used for total RNA purification. rRNA depletion was carried out using a Ribo-Zero Magnetic Gold (Yeast) kit (Illumina). An additional subtraction step was included to remove rRNA sequences from circularized cDNA as described previously (Spealman *et al.* 2016) with slight modifications. Subtractions were carried out in a 30 µL reaction volume, with 10 µL sample, 2 µL 20× SSC, and 2 µL of a rRNA subtraction pool containing custom-designed biotinylated oligonucleotides (Supplementary Table S1). The circularized cDNA was next used as a template for amplification of library PCR products. PCR products of 140–160 bp were recovered from excised gel slices, quantified by Agilent Bioanalyzer/Fragment analyzer and deep sequencing was performed using an Illumina Hiseq3000 machine at the Greehey Children’s Cancer Research Institute Genome Sequencing Facility (University of Texas Health Science Center at San Antonio). All ribosome profiling experiments were performed in biological triplicate.

### Re-annotation of the *C. albicans* transcriptome

In order to generate a comprehensive transcriptome annotation for *C. albicans*, we used version s07-m01-r27 of the General Transfer Format (GTF) file made available by the *Candida* Genome Database (CGD) (http://www.candidagenome.org/), which contains annotation of exons and coding domains in the Assembly 22 genome build of *C. albicans* SC5314. Previously described 5ʹ and 3ʹ UTR coordinates (Bruno *et al.* 2010), which were originally annotated using Assembly 21, were lifted over to the Assembly 22 build using the *liftOver* tool (Hinrichs *et al.* 2006) while the associated chain file was obtained from CGD. The lifted over candidate features were further filtered to ensure that there was no overlap with the existing coding domain sequences. In particular, if the lifted over coordinates of 5ʹ or 3ʹ UTRs overlapped with the coding domain, they were adjusted to ensure contiguity without any overlaps.

### RNA-seq and Ribo-seq data analysis

The quality of raw sequencing reads from RNA-seq and Ribo-seq datasets were assessed using FastQC (v 0.11.8) (Andrews 2010). Adaptor sequences and low-quality score (phred quality score < 5) bases were trimmed using TrimGalore (v 0.4.3) (Krueger 2012). Only reads of at least 20 nt. in length were retained. Trimmed sequences were mapped with STAR (v.2.5.2b) (Dobin *et al.* 2013) using Assembly 22 fasta as the reference and GTF s07-m01-r27 from CGD, allowing a mismatch of at most two positions. Only uniquely mapping reads were retained. All reads mapping to non-mRNA sequences were filtered out prior to downstream analysis. The corresponding non-mRNA sequences were also obtained from CGD (C_albicans_SC5314_version_A22-s07-m01-r27_other_features_no_introns.fasta). Periodicity analysis of Ribo-seq data was performed using ribotricer (v1.3.2) (Choudhary *et al.* 2020). For generating the list of potential ORFs to evaluate coding potential, we defined an ORF as any sequence with a start codon that differs from AUG by at most one nucleotide and has an in-frame stop codon. For each such potential ORF, ribotricer generates a score called the phase score (range 0–1) indicating the prevalence of the 1–0–0 pattern of Ribo-seq reads along the profile with higher scores indicating high similarity to an ideal Ribo-seq profile. Gene level counting was carried out using featureCounts (v1.6.4) (Liao *et al.* 2014). Since *C. albicans* Assembly 22 is a diploid assembly, the reads were counted for each allele separately (hapA and hapB) and then merged into a single read count per gene for differential expression (DE) and TE analysis. For ORFs, the Ribo-seq profiles of both the haploids were retained even if the ORFs across hapA and hapB represented the same sequence.

Differential expression (DE) analysis of RNA-seq data was performed using DESeq2 (Love *et al.* 2014). Only genes with at least one read per replicate were selected for performing the library size normalization step and running the moderated *t*-statistic test. We defined genes to be differentially expressed if the transcripts per million (TPM) was greater than 1 in at least two replicates for both 30°C and 37°C + serum conditions and if their absolute fold-change on a log_2_ scale was at least 1 with an FDR adjusted *P*-value of at least 0.05. To define differentially expressed genes at the Ribo-seq level, we used the same methods and criteria as in the analysis of RNA-seq data. Please note that one limitation of TPM is that it does not account for differences in transcript number (Zhao *et al.* 2020) and length between conditions. Differential TE analysis was carried out with riborex (Li *et al.* 2017) using only genes that had at least one read count per replicate across the two conditions in both Ribo-seq and RNA-seq samples. We defined genes to be exhibiting differential TE if their TPM was greater than 1 in at least two replicates for both 30°C and 37°C + serum conditions and their absolute fold-change on a log_2_ scale was at least 1 with a corresponding nonadjusted *P*-value at least 0.05. Volcano plots were generated using EnhancedVolcano (Blighe *et al.* 2020). Gene ontology (GO) and network analyses were performed with clusterProfiler (Yu *et al.* 2012) using the GO slim ontology file available from CGD. Please note that for each gene possessing potential uORF(s), uORF TE, and DE values represent the combined values for all potential uORFs in the 5ʹ UTR region. *C. albicans* genes possessing potential uORFs were defined as those genes in which both the Ribo-seq and RNA-seq uORF TPM was ≥1 in at least two biological replicates under all growth conditions.

### Identification of potential novel ORFs and uORFs

In order to identify potential novel ORFs, we performed reconstruction of the transcriptome using our RNA-seq and Ribo-seq samples from cells grown at both 30°C and 37°C in the presence of serum. More specifically, we used StringTie (v1.3.6) (Pertea *et al.* 2015) along with the GTF annotation file from CGD (r27) as the guide annotation (-G GTF) as well as the mapped reads. For each mapped experiment and the guide annotation, StringTie outputs a reconstructed transcriptome annotation with potentially novel transcripts using the mapped reads as evidence for expression. One potential disadvantage of StringTie is that in certain cases transcriptional noise may make it difficult to distinguish intron/exon boundaries. However, one advantage is that it allows for incorporation of both RNA-seq and Ribo-seq data in the transcriptome assembly. We then created a consensus catalog of novel transcripts by collapsing the novel transcripts obtained in each individual GTF to one representative transcript. For each novel transcript that was previously unannotated in the GTF file obtained from CGD, we searched for potential ORFs using ribotricer and allowing for all possible start codons with at most one nucleotide difference from AUG. Only the longest potential novel, ORFs were retained for downstream analysis. In addition, all potential novel and uORFs were filtered for those showing TPM ≥ 1 in at least two replicates for one growth condition.

### Identification of ribosome pausing sites

We developed a new method, ribopaus, to identify transcriptome-wide ribosome stalling sites using Ribo-seq data. Briefly, this method involves locating peak pileups in a smoothed profile of Ribo-seq data. A Savitzky–Golay filter (Savitzky and Golay 1964) was used to denoise the Ribo-seq pileup and then a *Z*-score approach was applied to identify peaks at sub-codon resolution. The Savitzky–Golay filter acts as a low-pass digital filter for smoothing the data. It finds a low-degree polynomial fit over adjacent points by the method of linear least squares and can increase the signal-to-noise ratio without distorting the signal overall. This is achieved by convolution, wherein subsets of adjacent data points are fitted with a low-degree polynomial by the method of linear least squares. An analytical solution exists for finding the solution to the least-squares problem if the data points are equally spaced (Savitzky and Golay 1964). For each candidate, ORF and the corresponding Ribo-seq profile as obtained from ribotricer, we applied the Savitzky–Golay filter. Peaks were then called such that the called site had a signal-to-noise ratio (*Z*-score) above 2.5, where the noise is estimated by fitting a single variance parameter for the entire profile. For each such peak, the corresponding *P*-value was calculated for a gaussian distribution whose mean and variance are empirically estimated from the given profile. Let $Y_{1:\;T}=\{y_{1},y_{2},...,y_{T}\}$ represent the profile of read counts over T codons. The Savtizky–Golay filter provides a smoothening approach that retains the shape of the read profile. It performs a convolution such that the denoised read counts at $t^{\text{th}}$ codon is given as:

$$y_{t}^{\prime }=\sum_{j=\frac{1-m}{2}}^{\frac{m-1}{2}}C_{j}y_{t+i},$$

where $\frac{m\;-\;1}{2}\leq t\leq T-\frac{m\;-\;1}{2}$. The coefficients $C_{j}$ are derived analytically. A $k^{\text{th}}$ polynomial is fit using linear least square to a set of m adjacent points where m is an odd number. We fit a polynomial using 15 adjacent points (m=15) here. Given codon positions {1,2,…, T}, we define a new variable z such that $z=t-\overset{-}{t\;}$where $\overset{-}{t}$ is the central point $\overset{-}{t}=\frac{\left(T+1\right)}{2}$. The polynomial is given as:

$$Y_{j}=a_{0}+a_{1}z+a_{2}z^{2}+\cdots +a_{k}z^{k},$$

where $Y_{j}=\{y_{j-\;\frac{m-1}{2}},y_{j-\;\frac{m-1}{2}\;+\;1},\ldots ,y_{j+\;\frac{m-1}{2}}\}$ represents a set of m adjacent read profile values centered at position j. The coefficients of this polynomial $a_{0},a_{1},\ldots ,a_{k}$ are obtained by $a={\left(J^{T}J\right)}^{-1}J^{T}Y_{j}$ where $a=\{a_{0},a_{1},\ldots ,a_{k}\}$ while J is a Vandermonde matrix with its $i^{\text{th}}$ row as values $1,z_{i},z_{i}^{2},\ldots$ In summary, the convolution coefficients $a_{0},a_{1},\ldots ,a_{k}$ are elements of the matrix $C={\left(J^{T}J\right)}^{-1}J^{T}.$ We used the implementation of the Savitzky–Golay filter in scipy (Virtanen *et al.* 2020) (signal.savgol_filter).

### Data availability

Strain available upon request. Supplementary files available at FigShare. Supplementary Material file contains a listing of all supplementary materials as well as Supplementary Table S1, listing of biotinylated subtraction oligonucleotides used for *C. albicans* ribosome profiling experiments, Supplementary Table S2, number of genes showing significantly altered TE and RNA differential expression during the *C. albicans* yeast-filament transition, Supplementary Figure S1, scatter plots showing consistency among Ribo-seq and RNA-seq biological replicates, Supplementary Figure S2, GO and network analysis of genes showing DE at the transcript level and Supplementary Figure S3, volcano plot of genes showing DE at the transcript level during the *C. albicans* morphological transition. Supplementary Data set S1 contains TE and RNA DE data for annotated genes as well as potential novel genes and uORFs in *C. albicans* cells undergoing the yeast-filament transition. Supplementary Data set S2 contains GO data for genes showing alterations in TE and RNA DE during the *C. albicans* morphological transition as well as *C. albicans* genes possessing potential uORFs and ribosome pausing sites. Supplementary Data set S3 is a re-annotated *C. albicans* GTF file. Supplementary Data set S4 is a GTF file for potential novel *C. albicans* ORFs only. Supplementary Data set S5 provides a listing of *C. albicans* potential uORFs identified in this study (please note that potential uORFs identified for both alleles are shown). Supplementary Data set S6 contains a listing of potential novel *C. albicans* ORFs identified in this study. Supplementary Data set S7 contains a listing of *C. albicans* ribosome pausing sites identified in this study. All raw and processed sequencing data generated in this study have been submitted to the NCBI Gene Expression Omnibus (GEO; https://www.ncbi.nlm.nih.gov/geo/) database under accession number GSE154488. All custom scripts used to generate data for this manuscript, including ribopaus, are available at Github: https://github.com/saketkc/2020_MCSK. A full description of the ribopaus method is provided on pp. 3-4. 

Supplementary material is available at figshare DOI: https://doi.org/10.25387/g3.13173215.

## Results

### Optimization of ribosome profiling for *Candida albicans*

Ribosome profiling (Ribo-seq) is a powerful approach that allows for genome-wide determination of translational activity (Ingolia *et al.* 2009, 2012; Brar and Weissman 2015). Briefly, cell lysates are treated with a protein synthesis inhibitor to halt translation and cells are lysed under conditions that maintain *in vivo* ribosome positions on mRNA. The lysates are treated with nuclease so that only mRNA sequences bound to the ribosomes remain intact. The mRNA “footprints” bound to ribosomes (RPFs) are then purified and ligated to single-stranded adaptors for cDNA synthesis, followed by PCR amplification and deep sequencing (Ingolia *et al.* 2012). By identifying all sequences bound to ribosomes on a genome-wide scale, ribosome profiling generates a complete picture of translational activity in an organism and, importantly, also serves to identify specific regions of ribosome stalling and translational inhibition (Shalgi *et al.* 2013; Marks *et al.* 2016; Barth *et al.* 2019; Riba *et al.* 2019).

A wild-type *C. albicans* strain was strongly induced to undergo the yeast-filament morphological transition and cells were harvested from both 37°C + serum (filament-inducing) and 30°C control (non-filament-inducing) cultures for both RNA-seq and Ribo-seq analysis. Using methods from a previous ribosome profiling study to determine allele-specific gene expression in *C. albicans*, as well as established Ribo-seq protocols in *S. cerevisiae*, we developed an optimized ribosome profiling approach for *Candida albicans* (Muzzey *et al.* 2014; Spealman *et al.* 2016), see *Materials and Methods*). Nuclease titration studies were used to identify an optimal RNase I concentration for digestion of ribosome-bound RNAs. Ribosomal RNA (rRNA) contamination presented a particular challenge in the preparation of *C. albicans* samples for ribosome profiling and appeared to vary with growth conditions.

**Figure 1:**
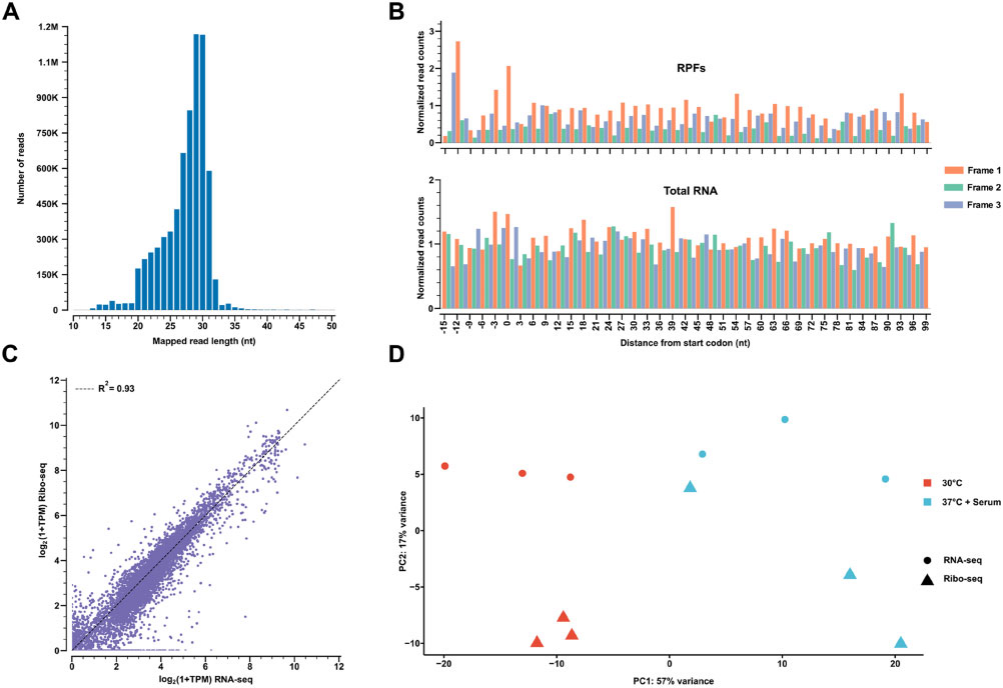
Validation of ribosome profiling data from *C. albicans*. (A) Example read length distribution plot for RPFs from a sample grown in YEPD at 30°C. (B) Examples of corresponding reading frame enrichment metagene plots for both RPF and total RNA samples generated using a 29 nt. read length. Normalized read counts for each position relative to the start codon (located at position zero) of the 5ʹ end of genes are shown. Characteristic 3-nucleotide periodicity is observed in the RPF plot. (C) Scatter plot showing a general correlation between Ribo-seq and RNA-seq read counts for the same sample described in part (A). (D) Principal Components Analysis (PCA) for RNA-seq and Ribo-seq samples grown in YEPD at 30°C and YEPD + serum at 37°C (all biological replicates shown). nt, nucleotide; TPM, transcripts per million.

A read length distribution plot indicated that *C. albicans* RPFs peaked in the range of 27–31 nt. (Figure 1A), which is consistent with that observed for other organisms. In addition, metagene plots demonstrated that RPFs, but not total RNA, reads showed a characteristic 3-nucleotide periodicity at the 5ʹ end of genes in the vicinity of the start codon, with a larger peak at the −12 position, indicating the P-site offset (Ingolia *et al.* 2009) (Figure 1B). All of our RPF samples displayed phase scores >0.41, indicating significant periodicity (see *Materials and Methods* for a description of the phase score). Overall, on a genome-wide level, we observed a strong correlation between RNA-seq and Ribo-seq read counts (Figure 1C), showing that most *C. albicans* genes which show high transcript levels are also translated. A principle components analysis (PCA) also generally showed distinct groupings for all three biological replicates of both RNA-seq and Ribo-seq samples for each growth condition (Figure 1D), as expected. Consistency among RNA-seq and Ribo-seq replicates was also verified by an observed strong correlation in read counts using all pairwise combinations (Supplementary Figure S1). Altogether, our optimized ribosome profiling approach yielded a robust dataset that can be used to determine a variety of translational parameters associated with the *C. albicans* morphological transition.

### Filament-induced transcripts involved in pathogenesis and virulence-related processes show reduced TE during the *C. albicans* morphological transition

**Figure 2:**
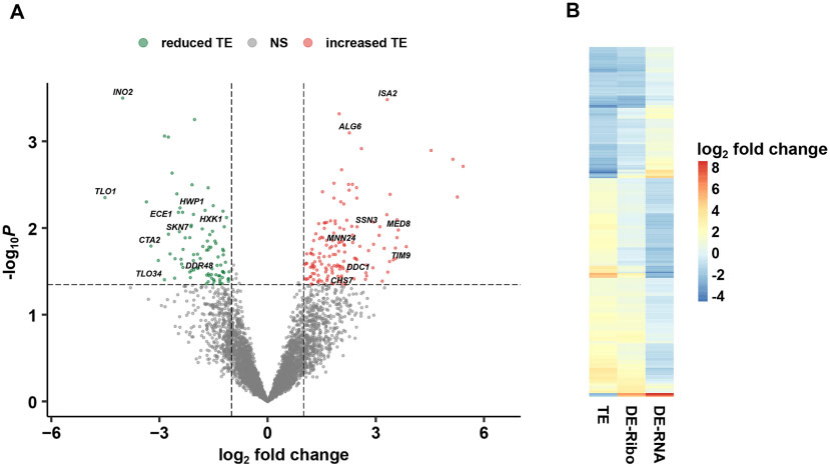
Identification of genes showing altered translational efficiency (TE) during the *C. albicans* morphological transition. (A) Volcano plot of *C. albicans* genes showing altered TE in the presence (37°C + serum) *vs* absence (30°C) of filament-inducing conditions. Vertical dotted lines indicate twofold change cutoff in TE. Horizontal dotted line indicates *P *=* *0.05 cutoff. Genes of interest are shown in black. (B) Heat map of genes showing altered TE in the presence *vs* absence of filament-inducing conditions as defined in part (A) and Supplementary Table S2. Corresponding differential expression (DE) values derived from both Ribo-seq (DE-Ribo) and RNA-seq (DE-RNA) in 37°C + serum *vs* 30°C are shown. NS, nonsignificant.

TE represents a key translational parameter that can be determined using both RNA-seq and Ribo-seq data (Ingolia *et al.* 2011; Ingolia 2016). Using a previously described method for determination of TE (Li *et al.* 2017), we identified 176 genes showing increased TE and 111 genes showing reduced TE during the *C. albicans* morphological transition (Figure 2A, Supplementary Table S2 and Dataset S1). A significant fraction of these genes showed large alterations (≥4-fold) in TE (Supplementary Table S2). Approximately half of all genes with increased TE also showed increased DE of Ribo-seq reads and decreased DE of RNA-seq reads during growth in serum at 37°C *vs* 30°C alone (Figure 2B). The remainder of these genes showed reduced DE for both Ribo-seq and RNA-seq reads with a larger reduction in DE for RNA-seq reads. An opposite expression pattern was observed for genes with decreased TE (Figure 2B).

**Figure 3:**
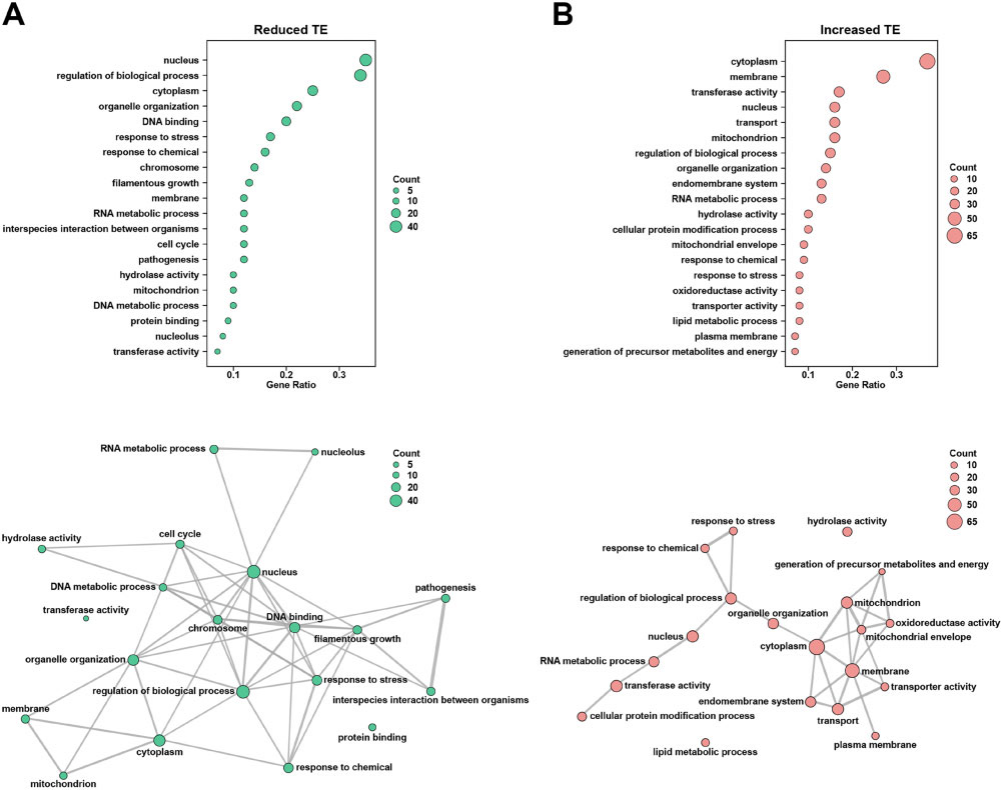
Gene ontology (GO) and network analysis of genes showing altered TE. (A) Genes showing significantly reduced TE as defined in Supplementary Table S2 were classified by GO terms using clusterProfiler (Yu *et al.* 2012) and the *C. albicans* GO Slim ontology (*Candida* Genome database, http://www.candidagenome.org/) (upper panel). Overlap among genes associated with specific GO terms is denoted by line thickness in a network analysis also carried out with clusterProfiler (Yu *et al.* 2012) (lower panel). (B) Genes showing significantly increased TE as defined in Supplementary Table S2 were classified by GO terms (upper panel) and GO terms were used for network analysis (lower panel) as described in part (A). Only GO terms with gene ratios ≥0.07 are shown. Count = number of genes within each GO term.

Among the set of genes showing significantly reduced TE during the *C. albicans* morphological transition, a GO analysis indicated strong representation of gene categories associated with filamentous growth, pathogenesis, hydrolase activity, response to stress, DNA-binding activity, and the cell cycle (Figure 3A and Supplementary Data set S2). A network analysis indicated a strong overlap among genes in gene categories associated with reduced TE (Figure 3A), suggesting that their expression may be controlled by a common translational mechanism(s); several GO terms represented hubs in this network, including DNA-binding, nucleus, chromosome and regulation of the biological process. Interestingly, many genes in several gene categories involved in pathogenesis and/or virulence-related processes were strongly induced at the transcriptional level, based on both previous studies (Nantel *et al.* 2002; Kadosh and Johnson 2005; Bruno *et al.* 2010), as well as our own RNA-seq results (Table 1 and Supplementary Data set S1). These genes included *HWP1*, which encodes a mammalian transglutaminase substrate mimic important for adherence to host cells (Staab *et al.* 1999), *ECE1*, encoding the candidalysin toxin essential for mucosal infection (Moyes *et al.* 2016), *DDR48*, which encodes a stress-associated protein (Dib *et al.* 2008) and *RBT1*, encoding a protein similar to Hwp1 (Table 1 and Supplementary Data set S1). Several genes encoding transcriptional regulators that control morphogenesis (*SFL2*), iron regulation (*SFU1*), and phosphatidylcholine/phosphatidylinositol biosynthesis (*INO2*) as well as signaling proteins important for both filamentation (*HXK1*) and the response to oxidative stress (*SKN7*) also were transcriptionally induced but showed significantly reduced TE (Table 1 and Supplementary Data set S1). In addition, this group of genes also included two members of the *TLO* (telomere-proximal) family, *TLO1* and *TLO34*. Although *TLO1* and *TLO34* have not yet been specifically characterized, many *TLO* gene family members encode Mediator complex subunits that have roles in *C. albicans* virulence and a variety of virulence-related processes, including morphogenesis, biofilm formation, the response to oxidative stress and antifungal drug resistance (Sullivan *et al.* 2015; Moran *et al.* 2019). Altogether, these findings suggest that many genes transcriptionally induced during the *C. albicans* morphological transition associated with virulence and pathogenesis are under tight negative translational control.

**Table 1:** Genes transcriptionally induced during the *C. albicans* morphological transition and involved in pathogenesis and/or virulence-related properties show significantly reduced TE

Gene name^a^	Ref. #	Description^a^	Fold change in transcript^b^	Fold change in TE^c^
*TLO1*	orf19.7544	Member of *TLO* gene family	15	−22.8
*INO2*	orf19.7539	Transcriptional activator of genes involved in phosphatidylcholine and phosphatidylinositol biosynthesis	3.4	−16.2
*C3_05990C*	orf19.7380	Protein of unknown function	17.8	−13.9
*CTA2*	orf19.6112	Putative transcription factor; Med2 mediator domain	15.2	−9.4
*TLO34*	orf19.2661	Member of *TLO* gene family	21.4	−7.3
*ECE1*	orf19.3374	Candidalysin, cytolytic peptide toxin essential for mucosal infection	388.0	−5.5
*HWP1*	orf19.1321	Hyphal wall protein, adhesin, host transglutaminase substrate mimic	362.0	−5.4
*SKN7*	orf19.971	Putative response regulator in phophorelay signal transduction; required for H_2_O_2_ resistance	3.7	−4.9
*DDR48*	orf19.4082	Immunogenic stress-associated protein	22.1	−4.5
*HXK1*	orf19.2154	GlcNAc kinase; required for hyphal growth and virulence	4.2	−4.3
*RBT1*	orf19.1327	cell wall protein similar to Hwp1; required for virulence	64.4	−2.7
*SFU1*	orf19.4869	GATA-type transcriptional regulator of iron-responsive genes; promotes gastrointestinal commensalism	2.0	−2.4
*SFL2*	orf19.3969	Transcriptional regulator of morphogenesis	2.0	−2.3
*SLK19*	orf19.6763	Alkaline-induce plasma membrane protein important for cell wall; required for virulence	2.2	−2.1

a Gene names and descriptions based on *Candida* Genome Database annotation (http://www.candidagenome.org).

b Indicates mean fold change in transcript levels (*n* = 3, TPM >1 in at least two replicates, *P*_adj_ ≤ 0.05) in cells grown in YEPD + 10% serum at 37°C *vs* YEPD at 30°C at the 1-h time point.

c Indicates mean fold change in TE (*n* = 3, TPM >1 in at least two replicates, *P *≤* *0.05) in cells grown in YEPD + 10% serum at 37°C *vs* YEPD at 30°C at the 1-h time point.

Gene categories showing strong representation in the set of genes with increased TE during the *C. albicans* yeast-filament transition included transport and transporter activity, membrane and endomembrane system, mitochondrion and mitochondrial envelope as well as lipid metabolic processes (Figure 3B and Supplementary Data set S2). A network analysis indicated significant overlap in this gene set among genes associated with transport, energy production, mitochondrial and/or membrane functions (Figure 3B). Genes associated with polyamine (*TPO5*), oligopeptide (*OPT8*) and manganese (*SMF12*) transport all demonstrated elevated TE (Table 2 and Supplementary Data set S1). In addition, genes encoding proteins associated with cell wall biosynthesis (*ALG6*, *MNN24*) mitochondrial protein maturation (*ISA2*), signal transduction (*SSN3*, *CEK1*, *PTC8*) and the physical process of filamentation (*CHS7*, *BEM1*, *RAX2*) all showed increased TE. Genes encoding several transcriptional regulators, including the *RPD3* histone deacetylase, involved in *C. albicans* white-opaque switching (Srikantha *et al.* 2001) and *HMS1*, important for morphogenesis (Shapiro *et al.* 2012), also showed a significant increase in TE (Table 2 and Supplementary Data set S1). Overall, these observations suggest that in addition to transcriptional mechanisms, *C. albicans* possesses specific translational mechanisms to promote processes associated with morphogenesis, including transport, cell wall biosynthesis, and energy production, during the yeast-filament transition.

**Table 2:** Selected genes showing significantly increased TE during the *C. albicans* morphological transition

Gene name^a^	Ref. #	Description^a^	Fold change in TE^b^
*MED8*	orf19.4497	Ortholog of RNA polymerase II mediator complex component	12.3
*ISA2*	orf19.6811	Protein required for maturation of mitochondrial [4Fe-4s] proteins	10
*DDC1*	orf19.245	Putative DNA damage checkpoint protein	7.6
*CWH8*	orf19.3682	Putative dolichol pyrophosphate (Dol-P-P) phosphatase	6.6
*SSN3*	orf19.794	Putative cyclin-dependent protein kinase	5
*ALG6*	orf19.1843	Putative glucosyltransferase involved in cell wall mannan biosynthesis	4.8
*MNN24*	orf19.1995	α-1,2-mannosyltransferase; required for normal cell wall mannan content	4.3
*CHS7*	orf19.2444	Protein required for chitin synthase III activity	4.1
*HMS1*	orf19.921	hLh domain Myc-type transcription factor required for morphogenesis	3.6
*TPO5*	orf19.151	putative polyamine transporter	3.5
*OPT8*	orf19.5770	oligopeptide transporter	3.5
*CEK1*	orf19.2886	ERK-family protein kinase; required for yeast-hyphal switch, mating efficiency and virulence	3.5
*PTC8*	orf19.4698	Predicted type 2C protein phosphatase; required for hyphal growth	3.1
*RPD3*	orf19.2834	Histone deacetylase; regulates white-opaque switching	3.1
*BEM1*	orf19.4645	Protein required for budding, hyphal growth, and virulence	2.6
*RAX2*	orf19.3765	Plasma membrane protein involved in establishment of bud sites and linear direction of hyphal growth	2.4
*SMF12*	orf19.2270	Manganese transporter	2.4

a Gene names and descriptions based on *Candida* Genome Database annotation (http://www.candidagenome.org).

b Indicates mean fold change in TE (*n* = 3, TPM >1 in at least two replicates, *P *≤* *0.05) in cells grown in YEPD + 10% serum at 37°C *vs* YEPD at 30°C at the 1-h time point.

As ribosome profiling requires the acquisition of standard RNA-seq data, we were able to re-examine transcriptional changes in gene expression during the *C. albicans* morphological transition as well. Compared to previous DNA microarray analyses (Nantel *et al.* 2002; Kadosh and Johnson 2005), we were able to identify much larger sets of genes that showed significantly increased (499) and decreased (387) transcript levels (Supplementary Table S2), which could be due to changes in transcription and/or mRNA decay rates. GO analysis indicated that among genes showing increased transcript levels, a variety of gene categories associated with *C. albicans* morphology and virulence, including filamentation, pathogenesis, response to stress, cell wall, cell adhesion, vesicle-mediated transport and biofilm formation were over-represented compared to the genome as a whole (Supplementary Figure S2B and Data set S3). Most genes in these gene categories formed a single network with interspecies interaction between organisms serving as a hub (Supplementary Figure S2B). Consistent with previous studies, many known filament-induced transcripts showed increased levels, including *ECE1*, *HWP1*, *DDR48*, the *CDC11* septin, *HGC1* cyclin-related protein, and the *UME6* transcriptional regulator (Supplementary Figure S3 and Data set S1). Additional genes of interest showing increased transcript levels included *CDR2*, a multidrug transporter, *EAF6*, a subunit of the NuA4 histone acetyltransferase complex important for filamentation, *ZRT2*, a zinc transporter, *CUP1*, a metallothionein involved in copper resistance, *ERG10*, a component of the ergosterol biosynthesis pathway and a variety of additional cell adhesin/cell surface proteins (including multiple members of the *ALS* and *PGA* gene families) (Supplementary Data set S1). Induction of these additional genes suggests that there may be new associations between the *C. albicans* yeast-filament transition and other biological processes, including stress resistance and metal homeostasis.

Among the set of genes showing reduced transcript levels during the *C. albicans* yeast-filament transition, gene categories involved in protein synthesis (ribosome biogenesis, ribosome, translation, translational regulator activity) were strongly over-represented compared to the genome as a whole and at least two of these gene categories were found in the same network (Supplementary Figure S2A and Data set S2). These genes included multiple members of the *RPS* and *RPL* gene families, encoding ribosomal subunits as well as genes in the *NOP* gene family, which is involved in ribosome biogenesis (Supplementary Figure S3 and Data set S1). *CAM1*, encoding a putative translation elongation factor, also demonstrated a lower transcript level. Other genes of interest showing reduced transcript levels included several transcriptional regulators controlling processes associated with antifungal resistance, such as *SUT1*, involved in sterol uptake, *STP4*, which is known to be induced in response to the echinocandin antifungal caspofungin and *ADA2*, important for cell wall integrity and sensitivity to caspofungin (Bruno *et al.* 2006) (Supplementary Data set S1).

### Identification of actively translating *C. albicans* potential uORFs and novel ORFs

**Figure 4:**
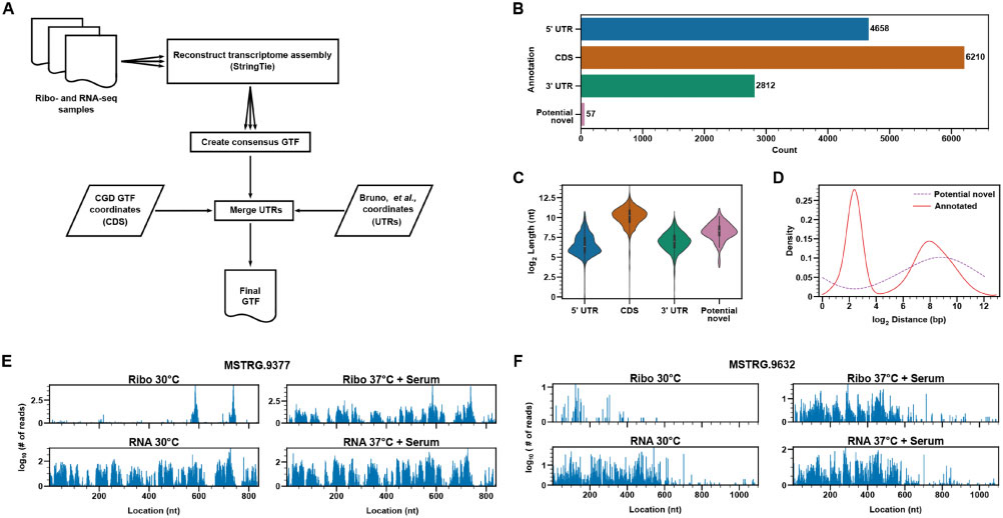
Identification of potential novel *C. albicans* ORFs. (A) Workflow for generation of a revised *C. albicans* transcriptome assembly that contains coordinates for both 5ʹ and 3ʹ UTRs. (B) Number of indicated transcriptome features identified based on the revised transcriptome assembly. (C) Violin plot showing length distributions for the indicated transcriptome features. (D) Plot showing distribution of intergenic distances between potential novel or known ORFs and adjacent annotated genes. (E) and (F) Ribo-seq (Ribo) and RNA-seq (RNA) read distributions under the indicated growth conditions for two potential novel ORFs. bp, base pair; CDS, coding sequence; nt, nucleotide.

The first step in identifying actively translated regions in the *C. albicans* genome involved generating a revised transcriptome annotation, as the previous annotation lacked certain key features, including the locations of both 5ʹ and 3ʹ UTRs. Using data from both RNA-seq and Ribo-seq samples, we reconstructed the *C. albicans* transcriptome to create a consensus transcriptome annotation (Supplementary Data set S3). This file was used as a basis to map gene coordinates from the *Candida* Genome Database (http://www.candidagenome.org) as well as coordinates for both 5ʹ and 3ʹ UTR locations that were reported previously (Bruno *et al.* 2010) (Figure 4A). Based on the revised transcriptome annotation, we define a significant number of 5ʹ and 3ʹ UTR regions as well as previously annotated coding sequences (Figure 4B). We were also able to identify 57 potential novel transcripts (Supplementary Data sets S4 and S6). As expected, the average length of 5ʹ and 3ʹ UTR regions was significantly shorter than that of coding regions (Figure 4C). The potential novel transcripts also exhibited a somewhat shorter average length compared to that of known genes. Interestingly, previously annotated coding sequences showed a bimodal distribution of intergenic distances to adjacent annotated genes (Figure 4D). Many potential novel genes also appeared to be located at a significant intergenic distance from previously annotated genes. These findings suggest that there are at least partial similarities in the genomic distribution of both potential novel and known ORFs in *C. albicans*.

Using a recently developed and powerful bioinformatics approach for detecting actively translating ORFs, ribotricer (Choudhary *et al.* 2020), we next identified on a global scale all actively translating regions in the *C. albicans* genome. Overall, we identified 1208 potential uORFs mapping to the 5ʹ UTR regions of 410 *C. albicans* genes, suggesting widespread translational regulation (Supplementary Data sets S1 and S5). A GO analysis of this gene set indicated a strong representation of gene categories associated with filamentous growth, response to stress, organelle organization, and the cell cycle (Supplementary Data set S2). Genes with potential uORFs included those involved in a variety of processes important for pathogenesis including morphology (*HGC1*, *CZF1*, *HMS1*, *RFG1*, *SHE3*), biofilm formation (*ROB1*), cell wall biosynthesis (*MNN21*, *BGL2*, *ANP1*), signaling (*HOG1*, *CCN3*, *RHO1*) as well as ergosterol biosynthesis (*ERG3*) (Supplementary Data set S1). Overall, we did not observe a clear anti-correlation between potential uORF TE and annotated ORF TE. In the set of genes showing reduced annotated ORF TE during the *C. albicans* yeast-filament transition, nine genes were identified as having potential uORFs in their 5ʹ UTR regions (Supplementary Data set S1). For two of these genes [*BAS1*, encoding a putative Myb-like transcription factor controlling purine biosynthesis (Wangsanut *et al.* 2017) and *GDS1*, encoding a putative mitochondrial protein induced in Spider medium biofilms (Nobile *et al.* 2012)] the potential uORF TE increased >2-fold upon serum and temperature induction. In the set of genes showing increased annotated ORF TE, 10 genes possessed potential uORFs (Supplementary Data set S1). Three of these genes [*CWH8*, encoding a putative dolichyl pyrophosphate (Dol-P-P) pyrophosphatase, *HMS1*, encoding a bHLH myc-type transcriptional regulator required for temperature-induced morphogenesis (Shapiro *et al.* 2012) and *PTC8*, encoding a predicted type 2 C protein phosphatase necessary for hyphal growth (Fan *et al.* 2009)] showed >2-fold reduced potential uORF TE during *C. albicans* morphogenesis. However, due to the short length of potential uORFs and their low abundance of read counts, potential uORF TE values are most likely not as accurate as those determined for known annotated ORFs.

All potential novel ORFs showed active translation (Supplementary Data set S6). Two examples of potential novel ORFs within mRNAs that are abundant under both filament-inducing (37°C + serum) and non-inducing (30°C) conditions and show increased translation under filament-inducing *vs* non-inducing conditions are shown in Figure 4, E and F. A few of the potential novel ORFs also showed significantly altered TE during *C. albicans* morphogenesis (Supplementary Data set S1). The large majority of potential novel ORFs are small in length (20 to 132 aa), which is not entirely unexpected given that larger ORFs have previously been annotated (http://www.candidagenome.org and Supplementary Data set S6). Given the short amino acid sequences, very few significant hits were identified for these putative proteins by a BLAST analysis. However, two of the potential novel ORFs (MSTRG.3487 and MSTRG.4032) showed 100% amino acid identity to hypothetical *C. albicans* proteins. Our data thus verifies that these ORFs do indeed encode actively translating proteins.

### Genes involved in *C. albicans* protein synthesis and cell wall functions possess ribosome pausing sites

**Figure 5:**
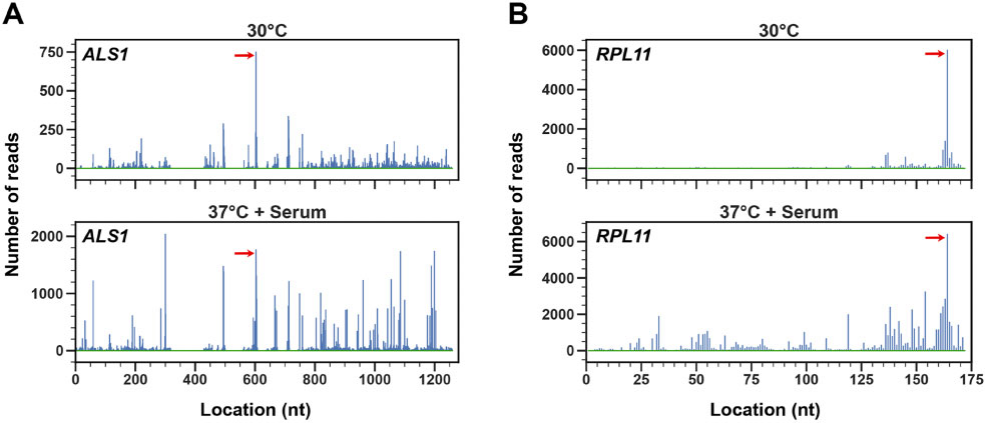
Identification of *C. albicans* ribosome pausing sites. RPF peaks identified from Ribo-seq data by ribosome pausing analysis for *ALS1* (A) and *RPL11* (B). Red arrows indicate consensus ribosome pausing sites based on three biological replicates across the indicated growth conditions. Please note that images depict read distributions from a single replicate. nt, nucleotide.

We have developed a novel bioinformatics method, called ribopaus, to identify ribosome pausing sites on a whole-genome scale from ribosome profiling data. Briefly, this method involves identifying RPF peak pileups using a smoothed profile of Ribo-seq data (see Materials and Methods section for details). Using stringent criteria, we were able to identify 25 features [uORFs, downstream open reading frames (dORFs), and annotated genes] showing consistent pausing sites in all three biological replicates in both the presence and absence of filament-inducing conditions (Supplementary Data set S7). A significant number of genes with ribosome pausing sites are involved in protein synthesis, including those encoding putative ribosomal proteins *RPL2*, *RPL11*, and *EFB1*, encoding translation elongation factor EF-1β, as well as *ASN1*, encoding a putative asparagine synthetase, *THS1*, encoding a putative threonyl tRNA synthetase and a putative tRNA-Arg synthetase (orf19.3341) (Supplementary Data set S7). Several cell wall/cell surface genes also showed clear ribosome pausing sites such as *ALS1*, an adhesin important for pathogenesis (Alberti-Segui *et al.* 2004; Zhao *et al.* 2004), *PGA14*, which encodes a putative GPI-anchored protein induced during cell wall regeneration, *LSP1*, encoding an eicosome component, *NCE102*, encoding a nonclassical membrane export protein as well as a potential membrane transporter (*orf19.6592*). Two examples of pausing sites identified by our analysis are shown in Figure 5 and a complete listing of pausing sites meeting our stringent criteria under both filament-inducing and non-inducing conditions is provided in Supplementary Data set S7.

## Discussion

While whole-genome global transcriptional profiling has been used in a variety of human pathogens to determine differential gene expression patterns associated with morphological transitions, biofilm formation and a variety of additional virulence properties (Nantel *et al.* 2002; Belland *et al.* 2003; Hwang *et al.* 2003; Kadosh and Johnson 2005; Murillo *et al.* 2005; Hautefort *et al.* 2008; Bruno *et al.* 2010; Silva *et al.* 2011; Upadhya *et al.* 2013; Tavares *et al.* 2019), very few studies have been carried out to determine the corresponding global translational profiles. Indeed, comparatively little is known about global translational mechanisms that control biological processes in a wide variety of genetic systems. Given that proteomic studies are not always accurate, sensitive or complete and that transcript levels can often serve as an inaccurate proxy for actual gene expression due to extensive translational regulation, there is a significant lack of knowledge regarding global changes in protein expression associated with virulence properties of human pathogens. The development of ribosome profiling, which has been used successfully in a variety of genetic systems, from yeast to humans, to study diverse biological processes including meiosis, stress responses and cancer (Brar *et al.* 2012; Brar and Weissman 2015; Blevins *et al.* 2019; Vaklavas *et al.* 2020), bridges this knowledge gap by providing comprehensive and accurate information about alterations in the global translational landscape at nucleotide-level resolution. Using this technique, Gilmore *et al.* (2015) demonstrated that several previously identified yeast- and hyphal-specific transcripts in the human fungal pathogen *Histoplasma capsulatum* also showed differential TE. Interestingly, a number of yeast phase-specific longer leader transcripts were identified that exhibited both transcriptional and translational repression and as many as half of all longer leader transcripts appear to be under translational control. To our knowledge, only a single previous study has used ribosome profiling in *C. albicans*, which was carried out to demonstrate coordinated allele-specific gene expression at both the transcriptional and translational levels (Muzzey *et al.* 2014). While this study took advantage of the fact that *C. albicans* is a naturally occurring obligate diploid to examine evolutionary forces controlling allele-specific gene expression, cells were grown exclusively under non-filament-inducing conditions.

Our current study thus represents the first use of ribosome profiling to examine alterations in the *C. albicans* global translational landscape associated with a major virulence property, the yeast-filament transition. TE calculations are based on the ratio of Ribo-seq DE to RNA-seq DE and actual changes in protein levels are reflected in the Ribo-seq DE ratio (37°C + serum *vs* 30°C) (Supplementary Data set S1). In comparing Ribo-seq DE ratios for several genes with corresponding fold changes observed in a previous quantitative proteomics study of the *C. albicans* yeast-hyphal transition (Monteoliva *et al.* 2011), we have observed a general correlation, thus validating our approach.

One of the most interesting findings was that many transcripts which are strongly induced at the transcriptional level during the morphological transition show significantly reduced TE. In addition to morphogenesis, these transcripts are involved in a variety of key virulence-related processes, and include the adhesin Hwp1, a mammalian transglutaminase substrate mimic that can form covalent linkages with host cells, as well as the Ece1 candidalysin toxin (Staab *et al.* 1999; Moyes *et al.* 2016). Our findings suggest that a translational fine-tuning mechanism is in place to ensure that critical virulence factors are tightly expressed only under the appropriate filament-inducing conditions. In contrast, genes associated with mitochondria, energy production, transport, and membranes showed significantly increased TE during *C. albicans* morphogenesis. These processes are all important for rapid hyphal growth and our findings suggest that *C. albicans* has evolved specific translational mechanisms to ensure that the associated proteins are expressed at high levels during morphogenesis.

In addition to providing new insights into translational changes in gene expression associated with *C. albicans* morphogenesis, our RNA-seq analysis has expanded the list of genes showing alterations in transcript levels during this process, at least when compared to those generated by previous microarray studies (Nantel *et al.* 2002; Kadosh and Johnson 2005). As expected, many gene categories associated with virulence-related processes, including filamentation, pathogenicity, adhesion, and stress responses are significantly over-represented in the serum and temperature-induced gene set compared to their representation in the genome as a whole. Interestingly, however, transcripts associated with ribosome biogenesis and translational control were strongly over-represented in the set of genes showing reduced transcript levels during *C. albicans* morphogenesis. These findings are consistent with a previous report that the *C. albicans* translational machinery is transcriptionally down-regulated during the yeast-filament transition in macrophages (Lorenz *et al.* 2004). Interestingly, reduced transcript levels of protein synthesis genes have also been reported for the reverse filament-yeast transition in response to depletion of *UME6* (Carlisle and Kadosh 2013).

Previous annotation of the *C. albicans* genome had classified many ORFs of less than 100 aa in length as “dubious” or “hypothetical” ORFs (*Candida* Genome database, http://www.candidagenome.org/). While RNA-seq results have helped to determine which of these putative ORFs are within abundant mRNAs, our ribosome profiling experiment is the first to provide functional evidence that several of these ORFs encode actively translating proteins. Although two of the putative novel ORFs identified by our study were 100% identical to such hypothetical proteins, the remainder did not show significant identity with other proteins. We believe this is largely due to the difficulty in obtaining significant BLAST hits with short (<100 aa) proteins as well as the fact that our method was able to detect proteins translating from noncanonical translation start sites in addition to standard AUG codons. Several of the potential novel ORFs identified by this study are likely to provide new insights into translational control of *C. albicans* morphology and pathogenesis. Indeed, a number of these potential ORFs showed alterations in transcription or TE during the *C. albicans* yeast-filament transition.

Our identification of over 1200 potential uORFs in the 5ʹ UTR regions of over 400 *C. albicans* genes suggests widespread translational regulation. Because many of these potential uORFs are located in 5ʹ leader sequences of genes associated with key virulence-related processes such as biofilm formation, morphogenesis, cell wall biosynthesis as well as those involved in antifungal resistance, translational regulation is likely to play an important role in controlling *C. albicans* pathogenesis. Consistent with this notion, several previous studies have implicated 5ʹ UTR regions in controlling two important transcriptional regulators of *C. albicans* morphology and virulence as well as a key regulator of white-opaque phenotypic switching and cell fate determination (Childers *et al.* 2014; Guan and Liu 2015; Desai *et al.* 2018). While we found no clear general anticorrelation between potential uORF TE and annotated ORF TE, our observation that several genes with alterations in annotated ORF TE during *C. albicans* morphogenesis, including those encoding several transcriptional regulators and a signaling molecule, also show inverse changes in potential uORF TE suggests that uORF-mediated translational mechanisms may be associated with the *C. albicans* yeast-filament transition. A very recent study in *Cryptococcus neoformans* has identified thousands of uORFs, several of which are actively repressing translation (Wallace *et al.* 2020), suggesting that uORF-mediated mechanisms may play an important role in controlling gene expression in a variety of fungal pathogens in addition to *C. albicans*.

In order to assess ribosome pausing on a global scale in *C. albicans*, we developed a new bioinformatics method, ribopaus. A previous method to accomplish this task relied primarily on deep learning, did not account for trends or heterogeneity and was trained only on human and yeast datasets (Zhang *et al.* 2017). Especially given the heterogeneity in RPF read distribution across transcripts, this method is more likely to result in false positive peaks. By contrast, our new method utilizes the dataset itself to create a denoised profile of the read counts. The denoising procedure utilizes information from nearby positions to create a smooth profile over each position such that the signal-to-noise ratio improves while the overall trend of read profile is maintained. *Z*-scores are then used to identify peaks at sub-codon resolution. We believe this new approach yields more reliable results compared to the previous method described by Zhang *et al.* (2017) and can be used for global identification of ribosome pausing sites in a wide variety of organisms and genetic systems. Using this approach, combined with stringent selection criteria, we were able to identify consistent ribosome pausing sites associated with at least 25 *C. albicans* genes. Interestingly, because a high proportion of these genes are involved in protein synthesis, components of the *C. albicans* protein synthesis machinery are themselves likely to be under tight auto-regulation at the translational level. Although we were able to identify sites that appeared to show differential pausing between yeast and filament-inducing conditions, the variance in differential pausing was too high to draw any conclusions.

Overall, our findings suggest that the *C. albicans* morphological transition, and most likely additional virulence-related processes in *C. albicans* and other fungal pathogens, is under significant translational control. Many, but not all, of these translational changes in gene expression do not simply parallel transcriptional alterations that have been previously observed. In addition to providing one of the first maps of the global translational landscape of a fungal pathogen and identifying several potential novel actively translating genes, our findings also suggest that a variety of key virulence processes in *C. albicans* are under tight translational control. Similar global translational mechanisms may be functioning to control diverse biological processes in other genetic systems. More specifically, our results are consistent with those of several previous ribosome profiling studies suggesting that global translational regulatory mechanisms are associated with important virulence processes in other human pathogens including *Plasmodium falciparum*, *Trypanosoma cruzi*, *Murine coronavirus*, and vaccinia virus (Caro *et al.* 2014; Smircich *et al.* 2015; Yang *et al.* 2015; Irigoyen *et al.* 2016). Given that several highly effective antibiotics are known to target bacterial translation mechanisms (Bhattacharjee 2016), our findings are likely to pave the way for future studies that develop novel antifungal therapies targeted against key translational control points.
