# Erratum to: Global translational landscape of the *Candida albicans* morphological transition

**DOI:** 10.1093/g3journal/jkab039

**Published:** 2021-04-21

**Authors:** Vasanthakrishna Mundodi, Saket Choudhary, Andrew D Smith, David Kadosh

This article was erroneously published in the January 2021 issue and has been republished in the February 2021 issue. The publisher regrets this error.

